# The gender responsiveness of social marketing interventions focused on neglected tropical diseases

**DOI:** 10.1080/16549716.2019.1711335

**Published:** 2020-01-20

**Authors:** Nathaly Aya Pastrana, Claire Somerville, L. Suzanne Suggs

**Affiliations:** a BeCHANGE Research Group, Institute for Public Communication, Università della Svizzera italiana, Lugano, Switzerland; b Gender Centre, Graduate Institute of International and Development Studies, Geneva, Switzerland; c Swiss School of Public Health+, Zurich, Switzerland

**Keywords:** Gender and Health Inequality, NTDs, infectious diseases, communicable diseases, behavior change, communication

## Abstract

**Background**: Gender is a determinant of health that intersects with other social stratifiers to shape the health and well-being of populations. Despite the recognition of gender in the global health agenda, limited evidence exists about the integration of gender considerations in interventions, including social marketing interventions, for the prevention and control of neglected tropical diseases. Social marketing is an ethical approach to behavior change aiming to benefit individuals, communities, and society. Since behaviors are gendered and affect disease transmission and healthcare patterns, one would expect social marketing interventions to be gender responsive.

**Objective**: This study aims to understand the extent to which social marketing interventions focusing on neglected tropical diseases are gender responsive.

**Methods**: This study uses data from social marketing interventions collected in a systematic review, this study examined 20 interventions addressing eight neglected tropical diseases in 13 countries. A modified version of the World Health Organization Gender Assessment Tool (GAT) was used to determine the gender responsiveness of the interventions, which was complemented by coding for intersectional sex and gender data. These results are presented in 12 themes.

**Results**: One schistosomiasis intervention implemented in China was assessed as gender responsive. It was not possible to answer many questions from the GAT due to limited data reported in the publications describing the interventions. Despite this, strengths and limitations were found in all the interventions in relation to the use of sex and gender concepts, the disaggregation of data, the consideration of environmental factors, and the involvement of women or men in the different stages of the interventions.

**Conclusions**: Many interventions showed positive actions towards gender responsiveness. However, only one was classified as gender responsive. Others failed to supply enough data for assessment. Recommendations about how sex and gender could be integrated into social marketing interventions are provided.

## Background

Neglected Tropical Diseases (NTDs) are prioritized in the 2030 Agenda for Sustainable Development (i.e. target 3.3) [1] and in the Beijing Platform for Action (i.e. C.90, strategic objective C.4 – action 109d, strategic objective E.5 – action 147f) [2]. These are diseases of poverty that affect more than a billion people globally [3,4]. NTDs ‘are disablers rather than killers’ [5,p.1] but can be fatal if untreated [6–10]. They impact health and socioeconomic development at the individual, household, and country levels [11]. Poverty and sociocultural factors, including gender, are some of the social determinants of health that are particularly relevant for NTDs [12], many of which disproportionately impact women, girls, and boys [13,14].

The way an NTD is transmitted and distributed, together with healthcare patterns, are all influenced by gender [15], as well as social stigma and discrimination associated with these conditions [16,17]. These gendered factors that shape the experience of disease are visible in neglected diseases such as leprosy [18], for which evidence shows that late diagnosis among women in comparison with men’s is attributed to gendered societal stigma, self-stigmatizing attitudes, the low status and economic dependence, and the lack of gender sensitivity of leprosy services [19].

Gender is socially constructed, varies over time, and is shaped by context [20–23]. Health systems, access, and behaviors are shaped by gender norms, roles, relations and intersect with other lines of inequity and discrimination [22–25] to shape the experience of populations and subgroups of people. The conjugation of gender with these social stratifiers generates barriers to access to opportunities, healthcare, and better wellbeing [22,26–28]. Addressing intersectional gender imbalances is, therefore, necessary to reduce health inequities [20].

Various bodies recommend addressing gender in societies’ structures [1,2,20,29,30]. For this, the collection and analysis of sex and gender data are necessary [31–33]. This starts, but does not end, with disaggregating data by sex (i.e. female, male, intersex), gender (e.g. non-binary), and other social stratifiers (e.g. age, socioeconomic status, race) [2,24,31,32,34]. The interpretation and use of disaggregated data are important. Failure to conduct gender analyses conceals patterns relevant to health outcomes [24,28,32].

Global and national policies recommend the use of Social Marketing to influence health behaviors [35–39]. Social marketing is founded on ethical principles and aims at influencing behaviors to improve the well-being of populations by combining marketing concepts with other approaches [40]. It mandates that problems and their determinants are well understood before strategies to address them are decided upon. Social marketing interventions adhere to a framework consisting of a series of criteria known as the ‘social marketing benchmarks’ [41,42]. These benchmarks distinguish social marketing from other approaches [43,44]. The benchmarks are: behavior-change, citizen orientation, theory, insight, segmentation, exchange/value, methods mix, and competition [42]. Over the years, the benchmarks have been updated. More recently, a new framework built on the benchmarks was created.

The Hierarchical Model of Social Marketing proposes that characteristics of social marketing interventions can be grouped into three categories [40]. The first is *principle*, which is about creating social value through exchange processes. The second category is named *concepts* and includes four elements: social behavioral influence, citizen/customer/civic orientation focus, social offerings, and relationship building. The last category, *techniques*, comprises five elements that are frequently but not solely used in social marketing interventions. The techniques are: integrated intervention mix, competition analysis and action, systematic planning and evaluation, insight-driven segmentation, and co-creation through social markets.

Social marketing interventions are designed to influence behaviors and deliver greater social good [40]. Contributing to equity is part of the core principles guiding the discipline [40]. Consequently, addressing gender inequities and inequalities should be intrinsic in social marketing interventions. Social marketing interventions could contribute to reaching gender equality goals in diverse ways. One could be by responding to the needs of populations based on their gender, that is, by being gender responsive. Understanding how gender interplays within a given setting and is associated with behavioral determinants, and their health outcomes is a prerequisite to having social marketing programs that are gender responsive. However, being gender responsive entails not just considering but also implementing measures to reduce the harmful effects of gender inequality and inequity that impede health outcomes [30].

The World Health Organization (WHO) prioritizes integrating gender into programs and policies [30,45]. It released a tool entitled ‘Human Rights and Gender Equality in Health Sector Strategies: How to Assess Policy Coherence’ [45]. The tool aims at improving the coherence among obligations and commitment of States towards human rights and gender equality, national frameworks (i.e. legal, policy, institutional), and national health sector strategies [45]. In addition to this tool, the WHO released the ‘Gender Mainstreaming for Health Managers Manual: A Practical Approach’ [30]. In this manual, the Gender Assessment Tool (GAT) was presented. The GAT focuses on determining whether programs and policies are gender responsive. While the manual also bestows the Gender Responsive Assessment Scale (GRAS), comprising five approaches for gender integration (i.e. gender-unequal, gender-blind, gender-sensitive, gender-specific, gender-transformative), the GAT does not assess the specific level applied by programs and policies.

### Rationale

Little is known about the integration of gender considerations in social marketing [46,47]. Similarly, despite recent calls to action, there is a dearth of literature examining the gendered dimensions of NTDs [15,48–50] and, to our knowledge, none that examine gendered dimensions of NTDs in social marketing interventions. Systematic reviews of social marketing health interventions have not included a gender responsiveness assessment [51–54]. Therefore, this study aims to understand to what extent social marketing interventions focused on NTDs are gender responsive.

## Methods

### Assessment process

**Figure 1.**
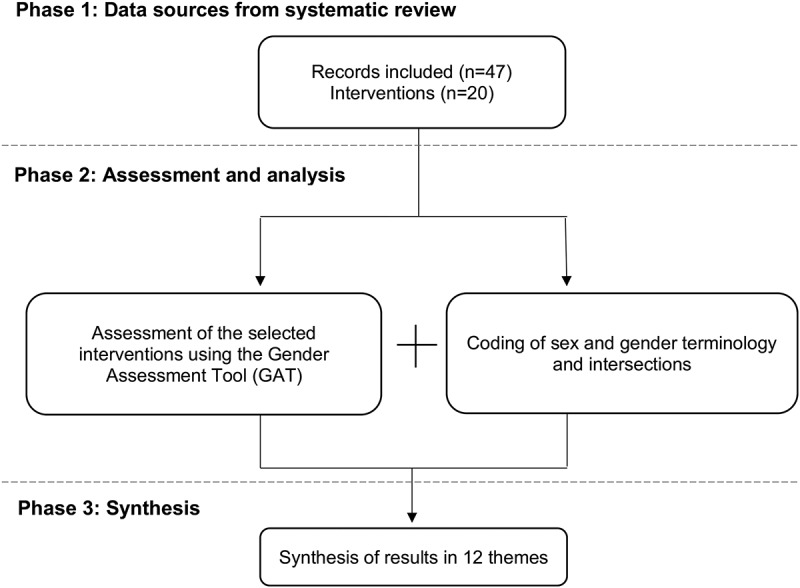
Assesment process

After obtaining data from a previous systematic review (see [55]), we conducted a gender assessment consisting of three phases (see Figure 1).

### Phase 1: data source

Data collected through a systematic review of social marketing interventions addressing neglected tropical diseases were used. Studies were eligible for inclusion if they were published between January 1991 and April 2017. They should have applied at least the social marketing concept ‘social behavioral influence’ and the technique ‘integrated intervention mix’, which are common in social marketing interventions [51,56–60]. The technique includes the traditional marketing mix, also known as the Ps (e.g. product, price, place, promotion, policy, partnerships), and other strategies such as public relations and community mobilization. The systematic review results, PRISMA flow diagram, and PRISMA checklist are reported elsewhere. Methodological guidelines are detailed in the research protocol registered with PROSPERO (see: CRD42017063858) [55].

#### Characteristics of the interventions

The social marketing interventions were implemented and evaluated between 1985 and 2013 (see Table 1). They focused on eight NTDs: cysticercosis (n = 1), dengue (n = 7), guinea worm disease (n = 2), leprosy (n = 1), lymphatic filariasis (n = 3), schistosomiasis (n = 4), soil-transmitted helminths (n = 1), and trachoma (n = 1). They were carried out in 13 countries; Australia [61–63], Brazil [64,65], China [66–70], Colombia [71], Honduras [72,73], India [74], Indonesia [75], Mexico [76,77], Nigeria [78–80], Saudi Arabia [81], Sri Lanka [82–84], Tanzania [85], and the USA [86].

**Table 1.** Intervention characteristics

Neglected Tropical Disease	Intervention Number	References of publications describing the intervention	Country	Year implemented	Funding provided by
Cysticercosis	**2**	[66,89,90]	China	2011–2013	Not available.
Dengue	**3**	[64,65]	Brazil	2012–2013	UNICEF/UNDP/World Bank/WHO Special Programme for Research and Training in Tropical Diseases (TDR). Additional grant from International Development Research Centre (IDRC), Ottawa, Canada.
	**11**	[71]	Colombia	NA	COLCIENCIAS Young Researchers and Innovators Program.
	**12**	[84,106]	Sri Lanka	2009–2010	Phase I: Special Programme for Research and Training in Tropical Diseases at WHO, WHO’s Regional Offices for South-East Asia and the Western Pacific, and the EcoHealth Programme of the International Development Research Centre (IDRC) of Canada. Phase II: Information not available.
	**13**	[81,104,105]	Saudi Arabia	2005–2006	National NGO “Zamzam” provided funds for materials.
	**16**	[76,77,101,102]	Mexico	1990	The Rockefeller Foundation, Health Sciences Division.
	**17**	[72,102]	Honduras	1990	Rockefeller Foundation, Health Sciences Division.
	**19**	[73]	Honduras	1996	Rockefeller Foundation, Health Sciences Division through the Integrated Dengue Control Project of the Ministry of Public Health of Honduras.
Guinea worm disease	**1**	[79,80,88,103]	Nigeria	1985–1986	UNDP/WorldBank/WHO Special Programme of Research and Training in Tropical Diseases, Social and Economic Scientific Working Group.
	**15**	[78]	Nigeria	NA	Information not available.
Leprosy	**8**	[82,83,108,122]	Sri Lanka	1990–NA	Ciba-Geigy Leprosy Fund, later known as Novartis Foundation for Sustainable Development (NFSD).
Lymphatic filariasis	**4**	[74]	India	2002	State funds and funds by the UNICEF/UNDP/World Bank/WHO Special Programme for Research and Training in Tropical Diseases.
	**6**	[86]	USA	2003–2004	US Department of Interior, the Council of State and Territorial Epidemiologists, the Pacific Island Health Officers Association, Research Corporation of the University of Hawai, PacELF and CDC’s Emerging Infectious Diseases Program.
	**7**	[75]	Indonesia	2002	Deutsche Gesellschaft für Technische Zusammenarbeit (GTZ) SISKES Project, Kupang, Indonesia.
Schistosomiasis	**9**	[85]	Tanzania	2002	Sida/SAREC, Stockholm and Skaraborg Institute for Research and Development, Skövde.
	**14**	[67]	China	1992–2003	Joint Research Management Committee of the World Bank Loan Project for schistosomiasis control in China and the Aid Group of schistosomiasis study for China and Southeast Asian, Japan.
	**18**	[68]	China	2000	UNICEF/UNDP/World Bank/WHO Special Programme for Research and Training in Tropical Diseases (TDR).
	**20**	[69]	China	1996	UNDP/World Bank/WHO Special Programme for Research and Training in Tropical Diseases (TDR).
Soil-transmitted helminths	**10**	[70,100]	China	2010–2011	UBS Optimus Foundation.
Trachoma	**5**	[61–63,91–99]	Australia	2010–2012	- Funding from private benefactors. - Trachoma Story Kits received support from private donors, Harold Mitchell Foundation, Ian Potter Foundation CBM Australia, Cybec Foundation, Aspen Foundation.

### Phase 2: assessment and analysis of the interventions

#### Gender assessment tool (GAT)

The WHO GAT [30] was used to determine the gender responsiveness of the interventions. The GAT consists of 23 ‘*yes*’ and ‘*no*’ response options and posits that if the majority of the answers of the first 18 questions are ‘*yes*’, the intervention could be considered gender responsive; and if the majority of answers to questions 19–23 are ‘*yes*’, the intervention could be considered as not gender responsive.

This tool was modified for the purpose of this study. Specifically, we removed question seven: ‘Do both male and female team members have an equal role in decision-making?’ because it could not be answered by the reviewer. Question number five asked if women and men participated in the design, implementation, monitoring, and evaluation stages, and so was divided into four questions so that each could be coded. Some words were also modified; for example, ‘policy or programme’ were replaced with ‘intervention’. ‘Target population’ was replaced with ‘public’ to consider the public not only as the primary target audience but also other people engaged with the intervention (e.g. community leaders) [55,87]. The GAT consists of three columns: (1) question, (2) yes, and (3) no. The adapted tool added two columns, one to document when data was insufficient to answer the question, and one to document quotes from the original publications describing the interventions, and reviewer notes.

The modified tool comprised 25 questions with three response options (yes, no, not available). Following the GAT guidelines, only the yes/no response options were considered in determining whether the interventions were gender responsive. The ‘*not available*’ responses from questions 1–20 from the modified tool were counted as ‘*no*’, and the ‘not available’ answers of questions 21–25 were counted as *‘yes*’. One researcher (the lead author), assessed each intervention using the GAT. Not available responses were kept disaggregated for reporting only.

An intervention was classified as *gender responsive* if questions 1–20 had at least 11 ‘*yes*’ responses. An intervention was classified as *not gender responsive* if there were at least four ‘*yes*’ responses to questions 21–25. Interventions that did not meet these minimum scores were not classified.

#### Sex and gender terminology and intersections

We also assessed the interventions for the use of concepts related to gender and sex. Considering intersections is important to understand the different experiences of varied groups of people. To complement the GAT, we added an additional search strategy to identify sex and gender and additional intersectional concepts across the dataset. A data table was used to assemble and code these concepts (see Table 2).

**Table 2.** Variables coded to examine intersectional sex and gender concepts

Variables
**Intervention ID**
**Target Audience**. *Response options: Woman, girls, men, boys, third gender, other.*
**Data disaggregated by sex and gender**. *Response options: Yes, to some extent, no, terms used.*
**Intersectional gender subgroup analysis conducted?** *Response options: Yes, to some extent, no.*
**Gender analysis conducted? (if not mentioned code as no)** *Response options: yes, no, comments.*
**Uses the word ‘sex’?** *Response options: yes, no.*
**Uses the word ‘sex’ to refer to biological characteristics (anatomy, physiology, genes, chromosomes, and hormones)?** *Response options: Yes, no, comments.*
**Uses these terms?** *Response options: Female (yes/no), male (yes/no), other (yes/no).*
**Uses the word ‘gender’?** *Response options: Yes/no*
**Uses the word ‘gender’ to refer to social constructions?** *Response options: Yes, no, comments.*
**Uses these terms?** *Response options: Woman (yes/no), wife (yes/no), girl (yes/no), husband (yes/no), boy (yes/no), man (yes/no), third gender or similar (yes/no).*
**Uses sex and gender to say the same thing?** *Response options: Yes (to refer to …); No; NA, only uses one word, which one?*
**The intervention shows understanding of the different ways people with intersecting forms of discrimination experience the diseases? If social stratifiers are used as (quantitative) variable not as ways of understanding the problem, mention it in the comments** *Response options: Race, ethnicity, sexual orientation, class, religion, age, disability, other – which?, none, comments.*

### Phase 3: synthesis in themes

Results from the GAT and from the intersectional sex and gender concept search were assembled into 12 themes. These themes were developed for this study through the process of data extraction and synthesis. The synthesis stage was an iterative process of reading and re-reading, extracting, and re-configuring until saturation of themes were agreed. This process helped identify recurrent and outlier findings, as well as potential quotes to include in the presentation of results. Table 3 presents the 12 themes with their corresponding data sources.

**Table 3.** GAT questions, intersectional sex and gender coding by theme

Theme	Question
1) Gender equality commitment	1. Do the vision, goals or principles have an explicit commitment to promoting or achieving gender equality?
2) Understanding of sex and gender	3. Does the intervention clearly understand the difference between sex and gender? Sex and gender data coded.
3) Selection of the publics	2. Does the intervention include sex as a selection criterion for the public?
	4. Does the public purposely include both women and men?
4) Participation of publics	5. Have women and men participated in the intervention *design* stage?
	6. Have women and men participated in the intervention *implementation* stage?
	7. Have women and men participated in the intervention *monitoring* stage?
	8. Have women and men participated in the intervention *evaluation* stage?
	9. Have steps been taken to ensure equal participation of women and men?
5) Stakeholders with gender expertise	14. Does the intervention include a range of stakeholders with gender expertise as partners, such as government-affiliated bodies, national or international non-governmental organizations or community organizations?
6) Data collection and reporting	12. Have methods or tools been piloted with both sexes?
	15. Does the intervention collect and report evidence by sex?
	16. Is the evidence generated by or informing the intervention based on gender analysis?
	18. Does the intervention include quantitative and qualitative indicators to monitor women’s and men’s participation? Sex and gender data coded.
7) Practical, strategic and health needs considered	11. Does the intervention consider and include women’s practical and strategic needs?
	17. Does the intervention consider different health needs for women and men?
8) Gender environment	10. Does the intervention consider the conditions and opportunities of women and men?
	13. Does the intervention consider family or household dynamics including different effects and opportunities for individual members, such as the allocation of resources or decision-making power within the household?
	19. Does the intervention consider gender-based divisions of labor (paid versus unpaid and productive versus reproductive)
	22. Does the intervention exclude one sex in areas that are traditionally thought of as relevant only for the other sex?
9) Understanding of public differences	21. Does the intervention exclude (intentionally or not) one sex but assume that the conclusions apply to both sexes?
	23. Does the intervention treat women and men as homogeneous groups when there are foreseeable, different outcomes for subgroups, such as low-income versus high-income women or employed versus unemployed men?
10) Communication	24. Do materials or publications portray men and women based on gender-based stereotypes?
	25. Does the language exclude or privilege one sex?
11) Addressing gender norms, roles and relations	20. Does the intervention address gender norms, roles and relations?
12) Intersectionality	Not related to a specific GAT question but to the overall findings. Intersectionality data coded.

## Results

### Gender assessment tool (GAT) results

According to the assessment conducted with the GAT, one of the 20 interventions was gender responsive [67]. This intervention focused on schistosomiasis in China and targeted schoolchildren and adult women and men. It aimed at reducing contact with snail infested water and increasing compliance with praziquantel-based chemotherapy. General and specific activities were implemented for each target according to their daily activities and roles. Although the intervention did not implement specific actions aimed at addressing gender inequities and inequality, it showed an understanding of the varied needs of its publics and reported results considering gender.

The data reported by many interventions were insufficient to answer some of the GAT questions (see Table 4). The average number of questions that were not possible to answer per intervention was 16 (64%), ranging from 6 [66] to 23 [69] questions. This lack of information was one of the reasons some were assessed as not being gender responsive. See Table 5 for the overall results of the GAT assessment.

**Table 4.** Gender assessment tool responses

Questions	Pattern of Responses		
	Yes	No	NA
	% (n)	% (n)	% (n)
Q1. Do the vision, goals or principles have an explicit commitment to promoting or achieving gender equality?	0,0% (0)	95,0% (19)	5,0% (1)
Q2. Does the intervention include sex as a selection criterion for the public?	35,0% (7)	25,0% (5)	40,0% (8)
Q3. Does the intervention clearly understand the difference between sex and gender?	0,0% (0)	60,0% (12)	40,0% (8)
Q4. Does the public purposely include both women and men?	25,0% (5)	35,0% (7)	40,0% (8)
Q5. Have women and men participated in the intervention *design* stage?	15,0% (3)	5,0% (1)	80,0% (16)
Q6. Have women and men participated in the intervention *implementation* stage?	90,0% (18)	0,0% (0)	10,0% (2)
Q7. Have women and men participated in the intervention *monitoring* stage?	0,0% (0)	0,0% (0)	100,0% (20)
Q8. Have women and men participated in the intervention *evaluation* stage?	0,0% (0)	5,0% (1)	95,0% (19)
Q9. Have steps been taken to ensure equal participation of women and men?	15,0% (3)	20,0% (4)	65,0% (13)
Q10. Does the intervention consider the conditions and opportunities of women and men?	25,0% (5)	15,0% (3)	60,0% (12)
Q11. Does the intervention consider and include women’s practical and strategic needs?	15,0% (3)	5,0% (1)	80,0% (16)
Q12. Have methods or tools been piloted with both sexes?	10,0% (2)	0,0% (0)	90,0% (18)
Q13. Does the intervention consider family or household dynamics including different effects and opportunities for individual members, such as the allocation of resources or decision-making power within the household?	15,0% (3)	10,0% (2)	75,0% (15)
Q14. Does the intervention include a range of stakeholders with gender expertise as partners, such as government-affiliated bodies, national or international non-governmental organizations or community organizations?	0,0% (0)	10,0% (2)	90,0% (18)
Q16. Is the evidence generated by or informing the intervention based on gender analysis?	5,0% (1)	15,0% (3)	80,0% (16)
Q17. Does the intervention consider different health needs for women and men?	10,0% (2)	0,0% (0)	90,0% (18)
Q18. Does the intervention include quantitative and qualitative indicators to monitor women’s and men’s participation?	20,0% (4)	10,0% (2)	70,0% (14)
Q19. Does the intervention consider gender-based divisions of labor (paid versus unpaid and productive versus reproductive)	15,0% (3)	10,0% (2)	75,0% (15)
Q20. Does the intervention address gender norms, roles and relations?	0,0% (0)	45,0% (9)	55,0% (11)
Q21. Does the intervention exclude (intentionally or not) one sex but assume that the conclusions apply to both sexes?	0,0% (0)	40,0% (8)	60,0% (12)
Q22. Does the intervention exclude one sex in areas that are traditionally thought of as relevant only for the other sex?	20,0% (4)	20,0% (4)	60,0% (12)
Q23. Does the intervention treat women and men as homogeneous groups when there are foreseeable, different outcomes for subgroups, such as low-income versus high-income women or employed versus unemployed men?	10,0% (2)	25,0% (5)	65,0% (13)
Q24. Do materials or publications portray men and women based on gender-based stereotypes?	5,0% (1)	5,0% (1)	90,0% (18)
Q25. Does the language exclude or privilege one sex?	5,0% (1)	5,0% (1)	90,0% (18)

Adapted [30]. Notes: N = 20 interventions. Data was not enough to answer this question = NA.

**Table 5.** Gender assessment tool results

	Number of ‘yes’ responses to:			
Intervention Number	Questions 1–20	Questions 21–25	Total number of questions not possible to answer, (% out of 25 questions)	GAT Assessment
1	6	4	7 (28%)	Not gender responsive
2	10	2	6 (24%)	Not possible to classify
3	1	5	20 (80%)	Not gender responsive
4	1	5	21 (84%)	Not gender responsive
5	1	5	21 (84%)	Not gender responsive
6	3	5	17 (68%)	Not gender responsive
7	8	4	12 (48%)	Not gender responsive
8	2	5	20 (80%)	Not gender responsive
9	3	5	19 (76%)	Not gender responsive
10	2	5	19 (76%)	Not gender responsive
11	2	5	20 (80%)	Not gender responsive
12	7	5	14 (56%)	Not gender responsive
13	1	3	14 (56%)	Not possible to classify
14	11	2	8 (32%)	Gender responsive
15	1	5	18 (72%)	Not gender responsive
16	8	0	9 (36%)	Not possible to classify
17	3	3	13 (52%)	Not possible to classify
18	2	3	20 (80%)	Not possible to classify
19	1	5	20 (80%)	Not gender responsive
20	0	5	23 (92%)	Not gender responsive

Scoring: Gender responsive if questions 1–20 had at least 11 ‘***yes***’ responses. Not gender responsive if questions 21–25 had at least 4 ‘yes’ responses. Not possible to classify if minimum scores were not met.

Nevertheless, all interventions had strengths and limitations with respect to how gender considerations were integrated into their actions (see Table 6). To understand the extent of integration of gender in the interventions, the following sections provide more specific insights into aspects that foster or inhibit gender responsiveness.

**Table 6.** Strengths and limitations of the interventions in relation to gender responsiveness

NTD	ID	Country	Strengths	Limitations
Cysticercosis	**2**	China	Some data disaggregated by sex and interpreted considering gender. Gender-specific focus groups. Consideration of some intersections (i.e. age/geographic location). Formative research identified villagers’ preferences for segmentation. Considered practical and health needs of women.	Intersections that emerged in the formative research were not visible in the intervention design nor in the presentation of results. The overall results data were aggregated. Used ‘manpower’.
Dengue	**3**	Brazil	Used a framework that includes the involvement of women as an indicator.	Did not disaggregate data by sex. Associates the word gender with women. Participation of men unknown.
	**11**	Colombia	Disaggregated the sex of the overall family members participating.	Most of the data not disaggregated by sex.
	**12**	Sri Lanka	Gender analysis conducted. Some data disaggregated by sex. Heads of households participating in the intervention and control clusters had a similar distribution of male/female participants.	Baseline household survey with 82,7% male vs 17.3% female heads of household. Female/male participation in focus groups discussions (FGDs) and in key informant interviews not disaggregated by sex. Results from FGDs informed gender analysis. Focused on women guided by findings from formative research, but it is unknown to what extent the data included women’s voices due to low participation of women in the household survey and the percentage of women in FGDs is unknown. Focusing on women was beneficial for the project, but unintended consequences beyond the intervention on the gender order of the community not mentioned. Used broad terms to refer to some publics.
	**13**	Saudi Arabia	Some data disaggregated by subgroups of female participants (i.e. students, teachers, supervisors).	Most data not disaggregated by sex. Reasons for targeting females based on normative roles in the family and household. Recommended to improve the education of females.
	**16**	Mexico	Some data disaggregated by sex. A table shows female/male responsibility on specific types of mosquito production sites (e.g. men – tires, women – cans). Reasons for including mostly female participants in the open interviews provided. Provided one example showcasing intersections (gender-age). Men and women participated in designing pamphlets specifically for women, men and families/general audience. Pamphlets address the behavior focus without leveraging on gender stereotypes.	Most data not disaggregated by sex. Used broad words to refer to some publics. Provided insights into the occupation of women, not men. Formative research consisted of several studies. Open interviews mostly with women due to difficulty to reach men. Pre-intervention survey exclusively with women, no reasons provided for not including men. Number of male/female participants in community groups not provided. Use of parenting roles to describe who received door-to-door information. Post-intervention survey designed for women. Included question about years of schooling of the participant and her husband. Reasons for not including men not provided.
	**17**	Honduras	Men and women participated in community meetings.	Data not disaggregated by sex. Used broad words to refer to some publics Purposely targeted women responsible for child care and the household. Unclear if men participated in formative research, or pre-post intervention surveys.
	**19**	Honduras	Mentioned that cleaning washbasins is usually a woman’s responsibility.	Data not disaggregated by sex. Participation of men is not clear.
Guinea worm disease	**1**	Nigeria	Disaggregated some data by sex. Selection of water filters considering some practical needs of women. Provided possible explanations (e.g. access to money) of buying patterns of men/women. Acknowledgement of the product adding to the domestic burden of women.	Used the words man/male and women/female interchangeably. Recommends targeting husbands in future interventions due to their role of provider/protector.
	**15**	Nigeria	Presented social and economic consequences of the disease (e.g. disability, effects on household/community economy). Mentioned once the participation of a midwife in training program.	Data not disaggregated by sex. Privileged men in the use of words and descriptions of the problem. Described the type of occupation of men but says nothing about women. Used broad words to refer to some publics.
Leprosy	**8**	Sri Lanka	Formative research conducted with men and women.	Did not disaggregate data by sex. Uses broad words to refer to some publics engaged. Communication material portrayed women in association with beauty.
Lymphatic filariasis	**4**	India	The use of the pronouns ‘him’ or ‘her’ suggested that Filaria Prevention Assistants (FPA) comprised men and women.	Did not disaggregate data by sex. Mentioned that results from the KAP survey data were consistent across age groups and genders, but no details informing how that consistency was determined.
	**6**	USA	MDA coverage data collected and reported disaggregated by age group, gender, and village. Transparency in reporting by informing that most KAP survey participants were females because they were in the household at the time of visits. Transparency in informing who was not legible to participate in MDA (children < 2 years old, pregnant women and individuals with illness).	Used broad words to refer to some publics
	**7**	Indonesia	Data collected ensuring the participation of women and men. Some data disaggregated by sex and reported in a table and in the narrative explanation of results. Communication materials tested with men and women separately.	Some data not disaggregated by sex. Results showed possible gender implications that were not reported comprehensively (e.g. women considered as one of the causes of the disease).
Schistosomiasis	**9**	Tanzania	Some data disaggregated (girls/boys). Formative research collected data related to household chores of girls and boys. Results presented differences by gender.	Some data not disaggregated by sex.
	14*	China	Target groups (schoolchildren, women, and men) selected considering age and sex. Water-contact patterns considered. Data in tables and in the text disaggregated by target group. Some activities were standard for all groups. Others developed and differentiated for each audience, focusing on specific barriers by gender. Comprehensive understanding of gendered differences in infested-water contact behaviors, and of their implications on intervention results.	Used broad terms to refer to schoolchildren and did not disaggregate participation by sex. The age ranges of women and men were broad (16–60 years), differences for other subgroups (e.g. younger/older) not considered.
	**18**	China	Demographical data disaggregated by sex. Sex included as a statistical variable.	Most data not disaggregated by sex. Used broad words to describe publics
	**20**	China	Sex included as a variable at baseline behavior observation. Post-intervention survey disaggregated participants. Mentioned a statistical difference by gender.	Most data not disaggregated by sex. Used broad words to describe publics Comic book images and video portray two boys and no girls.
Soil-transmitted helminths	**10**	China	Disaggregated statistical data by girls/boys. Described some differences between girls and boys.	
Trachoma	**5**	Australia	Once explicitly mentioned that children (boys and girls) were engaged. The KAP survey intentionally did not include sex and Indigenous status. Radio program featured women.	Data not disaggregate by sex. Used broad words to refer to the target audience.

Notes: In rural China, to ‘da gong’ ^1^ means to maintain residence status in your own village while moving to an urban area to earn money [90]. MDA = Mass Drug Administration. *Gender responsive intervention

### Theme 1: gender equality commitment

One GAT question asked whether the vision, goals, or principles of interventions explicitly demonstrated a commitment towards promoting or achieving gender equality. None explicitly mentioned this, but the intervention addressing lymphatic filariasis in Indonesia showed some inclination, as seen in the following text [75]:

“The campaign also reached men and women equally, righting the previous gender imbalance in knowledge about the disease. The open nature of the communication strategy at the community level meant that men and women were both exposed to the same messages. This may have been the first time some women had seen a hydrocele and connected it with filariasis.” [75, p. 1738]

### Theme 2: understanding of sex and gender

At least 60% (n = 12) of the interventions failed to delineate difference between sex and gender. Eleven interventions used the word sex explicitly to mention parameters, demographic data or statistics (e.g. female/male participants) [61–63,66–71,75–80,88–102]. Seven mentioned the word gender explicitly [64,69,74,75,84,86,89,90]; two referring to social constructions [75,84] and three to refer to demographic or statistical data [69,74,84]. Eight interventions used only the word sex not gender [61–63,67,68,70,71,76–80,88,91,100,101,103]; four used only the word gender and not sex [64,74,84,86]; and four did not use either word [72,81–83,85,102,104,105].

The cysticercosis intervention used the word ‘sex’ in a table to present demographic statistics and ‘gender’ to specify the use of ‘gender-specific or mixed gender groups’ in data collection. It used the words female/women interchangeably, and in one instance, compared males with women [82,83]. Similarly, another intervention addressing schistosomiasis compared men with females [67].

Other words related to sex and gender used in the publications describing the interventions included female [64,66–68,71,72,75–81,84,86,88–90,101,103,105], male [64,66–69,75–80,84,86,88–90,101–103], woman/women [64,66,67,72–77,79,80,82–84,86,88–90,101–103], man/men [66,67,75–80,82–84,88–90,101–103], girl [61–63,66,70,79,80,82,83,85,88–91,100,103,105], and boy [61–63,69,70,85,91,100]. None of the interventions made references to a third sex or to gender diverse people. Some used the terms men/male [66,79,80,88–90] and/or women/female interchangeably [64–66,79,80,88–90]. Four interventions did not use these words interchangeably, instead they used the same words (e.g. female/male, woman/men) consistently along the publication(s) in most instances [67,75–77,84,86,101,102,106].

Some interventions used words related to gender roles and relationships like wife [73,75,79,80,88], husband [75–77,79,80,82,83,88,101,102], mother [76,77,81,101,102,104,105], maternal [81,104,105] or father [76,77,81,101,102,104,105]. A guinea worm intervention used woman/female/wives or men/male/husband to describe the experience of women and men or to present statistical results. For example: ‘ … It was interesting to note the general pattern that men bought the filter for their wives to use … ’ [79,p.14].

### Theme 3: selection of the public(s)

Seven interventions selected publics considering their sex or gender [66,67,72,75–77,81,84,89,90,101,102,104–106]. Four implemented some of their activities focused on a specific group based on gender [67,76,77,79,80,84,88,101–103,106], such as conducting interviews with women because their domestic role included collecting water [79,80,88,103]. The gender responsive intervention implemented some activities with all three target audiences (i.e. adult women/men, schoolchildren), and some other activities were differentiated [67]. In contrast, dengue interventions showed a tendency to focus on women or girls; reasons included their role in the household and communities. For example, an intervention in Saudi Arabia purposely targeted female students (future mothers), teachers, and supervisors of high schools [81,104,105]; and a community-based intervention in Honduras purposely tried to reach women employed within the household [72].

Five interventions purposely included women and men [66,67,75–77,82,83,89,90,101,102]. For example, the cysticercosis intervention included men and women in the formative research by conducting gender-specific focus groups [66,89,90], and another addressing lymphatic filariasis in Indonesia ensured that both women and men participated in interviews as the following text shows [75,p.1733]:

“ … Each interviewer was responsible for interviewing seven men and seven women and the fifteenth person from either gender to ensure an even gender distribution of interviewees.” [75, p. 1733].

### Theme 4: participation of publics

The involvement of the publics along different phases of the intervention was considered. Three interventions [66,72,76,77,89,90,101,102] had women and men involved in the intervention design. Although the cysticercosis intervention did not involve participants in deciding the overall stages of the intervention, it did, however, have a male local toilet building supervisor and householders (female and male) decide the design of their toilets [66,90]. The dengue intervention in Mexico did not include women and men in the formative research studies nor in the evaluation, but included them in the design of education and communication material [76,77,101,102]. An intervention in Honduras also focusing on dengue had community meetings with women and men where health committees were formed, but the percentage of women and men involved was not provided [72].

Ninety percent (n = 18) of the interventions had some form of female and male participation in the implementation phase [63,64,67,68,70–78,80,82–86,90,91]. Of these, seven used broad generic words to group participants or stakeholders (e.g. school staff, children). Thus, the type of participation of females and males was not clear [63,68,71,73,76–78,86]. None reported if women, girls, men, or boys participated in the monitoring and evaluation stages.

A GAT question inquired whether actions were implemented to ensure equal participation of women and men. Three interventions portrayed this characteristic [75–77,90]. One used gender-specific data collection methods in the formative research [89,90], another calculated the interview sample purposely to include women and men [75], and the other had community groups composed of men and women design education and communication material [76,77].

### Theme 5: stakeholders with gender expertise

None of the interventions provided information suggesting having partners with gender expertise, even though some received funds from organizations that have shown support to gender mainstreaming and programming, such as the WHO Special Programme for Research and Training in Tropical Diseases. See intervention funders in Table 1.

### Theme 6: data collection and reporting

A GAT question asked about the piloting of methods or tools with both sexes. Two interventions piloted data collection tools or materials [67,75]. In one, focus groups with women and men were conducted separately to test communication materials [75]. Another intervention tested the potential acceptability of water cloth filters for guinea worm control during interviews with women only [79]. The gender responsive intervention showed gender sensitivity along its processes and informed about activities for each of its target audiences, but when mentioning the pretesting of a questionnaire, it did not specify if it was done with both sexes [67].

The collection and reporting of evidence by sex was another GAT question. Four interventions collected data by sex or gender [66,67,85,86,89,90]. By sex for, example, by designing the tools to include sex as a variable (female/male) [86]. By gender, for example, by having gender-specific focus groups [66,89,90]. Twelve interventions reported data disaggregated by sex [66–71,75–77,79,80,84–86,88–90,100,101] and four considered gender when interpreting it [67,84,85,90]. The gender responsive intervention is an example of how data collection and reporting can be disaggregated by sex and gender [67].

Quantitative or qualitative indicators were used by 20% (n = 4) of the interventions to monitor female and male participation [67,75,84,86]. Most data of the gender responsive intervention were disaggregated by the three study groups (i.e. schoolchildren, women, men) [67].

Disaggregated data was presented quantitatively in tables [67,75,77,84,90] and/or within the text [67,68,70,71,75–77,79,80,84–86,88,100,101,106], some did it using the labels ‘sex’ [71,75,79,90,102] and/or ‘gender’ [84,106]. Six described differences between female and/or male participants qualitatively [67,76,77,79,84,85,88–90,101]. Both quantitative and qualitative disaggregation used the words women [67,76,77,79,84,88–90,101], men [67,76,77,79,84,88–90,101], male [67,68,75–77,79,84,86,88–90,101], female [67,68,71,75–77,79,84,86,88–90,101], girls [85] and/or boys [70,85,100]. In some cases, the use of these words was not consistent within the text, meaning that the interventions did not use only female/male or women/men. In the case of two interventions targeting schoolchildren, also referred to as pupils, data was not disaggregated into girls and boys [67,68].

The intervention focused on trachoma in Australia did not disaggregate data by sex and did not consider gender differences. As the following text shows, a knowledge, attitudes, and practices survey with clinic, school, and community settings staff, purposely did not include sex as a variable: ‘ … Identifying features such as name, job title, sex, age, Indigenous status or other characteristics were not required … ’ [62,p.36].

One intervention addressing dengue in Sri Lanka conducted gender analysis using data from focus group discussions (FGDs) and key informant interviews, but it did not report the number of participants nor their sex distribution [84]. Although two other interventions did not implement a gender analysis [64,90], one collected data using gender-specific focus groups and interpreted findings considering differences between women and men [89,90]; and the other used a five-indicator analytical framework to assess community participation that included an indicator to monitor the involvement of women. Despite using this framework, the intervention did not disaggregate data by sex, nor interpret findings based on gender, nor present data suggesting that the participation of men was monitored [64].

### Theme 7: practical, strategic and health needs considered

Practical needs are short term or basic day-to-day necessities (e.g. easily accessible clean water). In contrast, strategic needs are those related to the subordinate position of a group in comparison to other groups and has to do with the enjoyment of rights, power, control over resources and access to opportunities (e.g. pay job) [107]. Health needs are related to physical and mental health. Practical needs were considered by three interventions [76,77,80,88,90], strategic needs were not explicitly mentioned, and health needs were contemplated by two interventions [67,90].

One of the interventions addressing guinea worm disease in Nigeria considered the burden on women when identifying tangible solutions to filter water, as women would be tired after walking long distances to collect water [80]. It also acknowledged that the product offered (monofilament nylon water filter) did not reduce women’s burden to obtain water, as would having a village well [88].

An intervention in China promoting the building and use of household toilets to address cysticercosis considered data about women and men preferences for toilet placement and design:

“Convenience was mentioned many times in all focus groups, but especially in the female focus groups. ‘It would be quite convenient if we all had our own toilets at home.’ ‘Some people are used to going to bathroom at five in the morning. You have to get up that early to open the door for them.’ Privacy and cleanliness were also especially valued by women.” [89, p. 125]

When determining who implemented behaviors related to control of mosquito production sites, a dengue intervention in Mexico found that men, not women, were responsible for the management of tires:

“ … when women were asked about tires, many remarked that the tires were not theirs, so they could not dispose of them to prevent the accumulation of water. Although they often stated that they encouraged that tires be discarded, or tried to control them by putting in used motor oil, they did not believe that action on their part was appropriate. In this case, the appropriate target group for messages about tires was found to be men.” [77,p.405].

Two interventions considered the different health needs for women and men [67,90]. The cysticercosis intervention tested women in childbearing age for pregnancy to ensure a CT scan was not conducted [90]. The gender responsive intervention addressed health needs of adult women by stressing the negative effects of risky behaviors on pregnancy and infants. In the case of adult men, the intervention highlighted the benefits of examination and treatment [67]. Another intervention focused on lymphatic filariasis in American Samoa did not address health needs directly, but was transparent in informing who did not participate in the MDA, namely: children below 2 years old, pregnant women, and individuals with grave illness [86].

### Theme 8: gender environment

#### Conditions and opportunities

Five interventions considered the conditions and opportunities of women and men [75,79,80,84,85,88,90], and three family or household dynamics [67,79,80,88,90]. A dengue intervention in Sri Lanka that conducted a gender analysis mentioned that women were at home more regularly than men and that for cultural reasons, they played an important role in the lives of girls and boys [84]. The gender responsive intervention in China was grounded on the understanding that infested-water contact behavior was related to recreational activities among schoolchildren, household chores among women, and public activities among men [67]. A schistosomiasis intervention focusing on schoolchildren in Tanzania conducted formative research that informed about their weekend chores, and presented differences between girls’ and boys’ household activities:

“ … Both boys and girls wrote they wash their school uniforms and fetch water, but girls also help more with household chores such as preparing food and cleaning the house. Boys more often graze cows and goats while both boys and girls help their parents working on the farms … ” [85, p. 84]

The intervention addressing cysticercosis in China considered the influence of people who have emigrated to urban areas for work (‘da gong population’) on the decisions of the villagers that remain in their setting. Those who ‘da gong’ are women (e.g. work in factories) and men (e.g. work in construction) who are more capable of doing physical labor, and who continue to provide economic support to their families despite not being physically present [90].

An intervention focusing on Lymphatic Filariasis in Indonesia, although not precisely differentiating the conditions and opportunities of women and men separately, provided data of how participants perceived the disease as a problem [75, p. 1734].

#### Gender-based divisions of labor

According to the GAT, 15% (n = 3) of the interventions considered gender-based divisions of labor [67,76,77,79]. In one, village women were farmers and also responsible for water collection and treatment, whereas men were farmers and responsible for selling the produce and managing the money [79]. Similarly, in the gender responsive intervention, men were mostly in contact with infected water during productive activities (e.g. fishing, agricultural production), whereas women while performing household chores (e.g. washing clothes and utensils) [67]. In another intervention in Mexico, women were responsible for the health and care of the family and household, nothing was mentioned about men’s roles [101].

Four interventions excluded men in areas that are traditionally considered applicable for women [72,79,80,84,105], three of these interventions focused on dengue [72,84,105]. For example:

“In the Focus Group Discussions, women were identified as the key actors in the entire process of cleaning homesteads and solid waste management at household level. Women spend more time at home than men, especially during the daytime. Culturally, the mother is the key figure guiding children in their day-to-day practices as well as in children’s educational process. Therefore, project activities centred around women as their role in the community enabled them to be better contributors to the waste management system” [84, p. 484].

Another intervention focusing on dengue in Saudi Arabia decided to target females because of traditional roles attributed to them:

“The target population was female students, teachers and supervisors in high schools because control strategies for DF [Dengue Fever] focus on good practice inside the home, which is mainly the responsibility of females. In addition, female students can be good health educators for their parents, especially their mothers” [81, p. 1059].

### Theme 9: understanding of public differences

A GAT question inquired if an intervention excluded (intentionally or not) one sex and assumed the conclusions applied to males and females. It was found that eight interventions did not have this characteristic [67,68,72,75,76,80,81,90], but evidence of the other interventions was not enough to suggest the interventions collected data with one sex was applicable to both.

A dengue intervention in Mexico included mainly women in interviews and mentioned difficulties in obtaining data (e.g. safety issues). They also conducted a pre/post KBP survey with women only. The design of information and communication materials was done via meetings with male and female community members, and their sex distribution was not provided. The researchers referred broadly to the community and spoke about the generalizability of findings:

“ … almost no men were interviewed, although efforts were made to contact them. Fortunately, it was possible to verify during community meetings held as part of a community-based intervention in the subsequent months that most of the findings were generalizable.” [101, p. 384]

Moreover, based on what is reported in the publications describing two interventions [61,63,79,80], these treated women and men as homogeneous groups despite possible varied outcomes if observed by subgroups. For example, a guinea worm intervention in Nigeria targeting community families and householders had previous experience in the setting and understanding of the local context. It did not develop different strategies for reaching village women versus town women, nor for approaching village women versus village men. Differences between these groups were considered at the end when reflecting on the results, but not in the stages of design, implementation, monitoring, and evaluation [79,80,88].

An intervention addressing trachoma in Australia had varied target audiences, namely children, and clinic, school, and community staff. Different activities were implemented with communities to reach children; and with clinic, school, and community staff to improve skills. These publics, however, were treated as homogeneous groups; for instance, subgroups were not considered (e.g. girls vs. boys, female/male community staff) [61,63].

### Theme 10: communication

#### Gender based stereotypes

Most interventions (n = 18) did not provide much detailed information about the portrayal of men and women in their communication materials or publications. Interventions that did provide some descriptions were aimed at dengue and leprosy.

The dengue intervention took place in Mexico and used community meetings to develop with the target audiences, pamphlets for women, men, and families:

“ … For example, in community meetings, men stated that messages should focus on the potential lethality of dengue fever … Based on this, and the fact that men have primary responsibility of tires, it was decided that an appropriate message for the target group of men would be ‘The tire which is in your backyard or workshop can cause the death of someone in your family’” [77,p.408].

“ … in the pamphlet produced for women. The front of the pamphlet showed a woman standing under a tree beside a flower pot and a vase in the window of a house. The caption at the top said ‘The mosquitoes which give us dengue can reproduce inside our houses.’ The woman was responding to the caption by saying ‘Don’t mosquitoes come from the underbrush?’ The pamphlet then went on to explain that although adult mosquitoes may rest in the underbrush, they can only reproduce in receptacles containing water … ” [77, p. 408]

The leprosy intervention in Sri Lanka focused its messages on what the publics valued: getting married and social acceptability. As can be observed in the following text, the association of beauty with women is emphasized:

“The television presentation depicted a young beautiful girl who has been cured of leprosy. It began with her getting ready for her wedding ceremony and being surrounded by her husband, mother-in-law, relatives and friends. It ended with her having a beautiful baby (personal communication). Another television scene showed a beautiful actress bathing in the river, when she suddenly dropped the piece of soap in her hand due to numbness from leprosy. This was followed by the campaign line ‘Go to the clinic for treatment’ … ” [108, p. 313]

#### Language

The language used in the publications describing the interventions was observed to identify if it excluded or privileged one sex. The pamphlets designed for a Mexican dengue intervention did not exclude nor privileged a group based on gender [77]. On the contrary, the wording used in a paper describing a guinea worm disease intervention implemented in Nigeria had a male bias (man = human beings):

“Man is the only significant reservoir of infection and control efforts are directed at him. Control can focus on man’s two behaviors – the drinking of water containing infected Cyclops and the exposing of ulcers to drinking water sources … ” [78, p. 265]

The cysticercosis intervention in China, although not explicitly excluding nor privileging one sex, used the word ‘manpower’. This word was associated with being physically capable of performing some activities [79]

### Theme 11: addressing gender norms, roles and relations

None of the interventions mentioned conducting activities to address gendered norms, roles, and/or relations. In fact, some focused on dengue provided information that reflected the contrary, that activities were leveraging on existing gendered patterns or normative expectations to reach the interventions’ goals [76,77,81,84]. For example, the dengue intervention in Sri Lanka focused activities on women because traditionally, their role within the household (e.g. caregiver) would benefit the intervention waste management activities [84].

Other interventions recommended focusing on women/girls [105] or men [79] in future interventions. The reasons were grounded on gender roles within the household and access to economic resources.

To understand a purchasing pattern in which men acquired the filter but their ‘wives’ were the users, a guinea worm intervention in Nigeria considered three possible explanations. One was that the majority of the salesforce was composed of men, who probably found it easier to approach other men. However, this did not correspond to the experience of a male research assistant who equally reached men and women, and still had more ‘husbands’ purchasing. A second possibility was that because the filters were innovative, the ‘husbands’ as heads of the household were in charge of introducing them to the family [79]. The third explanation was that they missed differences within (town women vs. village women) and between (women vs. men) genders, the latter in relation to the village male role of being the protector and provider of the family [79].

### Theme 12: intersectionality

Subgroup analysis based on the intersection of gender with other social stratifiers was not explicitly mentioned by any of the interventions, but some showed some consideration of how the experience of varied groups of people varied according to their gender [67,76,77,79,80,88,90,101], geographical location [79,80,88,90], status in life [90], age [67,90,101] and occupation [67]. One mentioned disability as a consequence of guinea worm disease and its effects on households and communities, but did this very broadly [78]. Another presented an example of how beliefs of older women may be different from those of middle-aged people, but did not provide more information to suggest intersections were considered [101]. The intervention addressing guinea worm in Nigeria explained reasons for which men purchased more filters than women and showed some understanding of differences based on geographical location and income generation (e.g. village/town women) [79].

The intervention focusing on cysticercosis in China showed sensitivity to the intersection of social stratifiers [90]. During formative research exercises village members were asked to segment themselves. Participants considered that segmenting villages according to their family names was meaningful, as well as by age and/or position in life (e.g. farmer, school age, middle-age staying, middle age da gong, old, older that stay, age 16 and under-students). The ‘da gong’ population comprises men and women able to perform labor-intensive jobs in urban settings while sustaining residency status in their own villages. Segmentation based on the location of the houses within the villages was also suggested by the villagers. Nevertheless, it is unclear whether the intervention used these segments suggested by the villagers because evaluation results are presented as a full sample. This intervention also considered cultural aspects (e.g. language, ethnicity, family values) in the selection of team members, data collection, and interpretation.

## Discussion

This study shows the extent to which social marketing interventions focusing on behaviors for the prevention and control of neglected tropical diseases are gender responsive. According to the GAT, only one intervention was gender responsive. The absence of an explicit commitment was clear in the interventions which is consonant with The Global Health 50/50 Report [34] that evaluated the gender responsiveness of 140 organizations working in global health, including the NTDs community, and found that about half of them did not explicitly express this commitment.

Overall, data reported in publications describing the interventions were not sufficient to respond to many GAT questions, resulting in a not gender responsive categorization. Nonetheless, interventions had strengths and limitations, and the qualitative analysis provides insights that we turn into recommendations about how sex and gender could be better integrated into the different stages of health interventions.

### Strengths and limitations of interventions

Interventions strengths, some of which were implemented by only one intervention, included disaggregating data by sex to a certain extent, calculating participant sampling to ensure men and women were included, providing narrative descriptions of the gender environment and their effects on behavior of participants, designing or testing communication material with men and women, and segmenting and implementing varied strategies for each segment, considering the different ways in which each segment was exposed to the risk behavior.

Limitations included not disaggregating data by sex, using gender-biased language, or sex and gender words that did not facilitate understanding if they were referring to biological characteristics or social constructions, and using broad words to refer to some publics which blurred the participation of men or girls and boys in some cases. The use of gender sensitive data collection tools and methods was limited, as well as the use of gender analysis to interpret and report findings. Some interventions failed to mention the quantity of females or males involved in different stages, and others made recommendations that perpetuated gendered roles. Dengue interventions that focused on the reduction of mosquito breeding places, tended to focus on women due to their responsibilities within the household or in their communities.

Gender is a relational, historical, and cultural determinant of health. The role of culture is fundamental in shaping the health trajectories of people based on their sex and gender. The interventions assessed in this study took place in 13 countries with different systems, some of which are more patriarchal. This is the case of dengue interventionsimplemented in four Latin American countries, in Sri Lanka and Saudi Arabia. The latter country has emphasized the ‘empowerment of men and the domestication of women’ [109, p. 1681]. An intervention alone cannot change how a system functions and should be culturally sensitive, but this does not imply that it cannot contribute with actions to avoid perpetuating gender inequities.

This study found that some interventions excluded men in areas traditionally associated with women, some leveraged on the gender order of the context in which they were operating, and others made recommendations that perpetuated gender imbalances. These are characteristics of gender unequal (perpetuate unbalanced gender constructions) and gender blind (ignores differences) approaches that are classified as not gender responsive [25,30,110,111]. The Global Health 50/50 Report [34] also found that the organizations assessed tend to be gender-blind and lack gender responsive programs.

Despite global calls for sex-disaggregated data and gender data [2], and the existence of tools to facilitate the collection and analysis of gender statistics [31,112], even the interventions more recently implemented were weak on this. The use of sex and gender related words also reflected a lack of understanding of their foundational concepts and their intersection with other social stratifiers. In this sample, the data continues to be binary focus (female/male, woman/man), and the absence of a third sex (i.e. intersex) and gender diversity (e.g. third gender, LGBT) was evident. While data availability may be partially determined by local systems and may vary depending on the income level of a country; it is suggested that future studies collect non-binary data and consider intersections with other social stratifiers. Other studies have also raised the lack of clarity in the use of sex and gender concepts and the reliance on binaries [24,25,28,32,33]. Findings also show that little is reported about the content of communication materials and of data collection tools used, which limits understanding of how these have responded to the local context and their possible effects.

### Recommendations for interventions

Any intervention can contribute to gender equality by having it as a primary goal or by avoiding the perpetuation of inequalities based on intersectional gender. To better integrate sex and gender into the different stages of global health interventions and consequently be more gender responsive, we propose the following recommendations that cross-cut the stages of formative research, design, implementation, monitoring, evaluation, and reporting of interventions.

In all stages, but particularly during the formative research that informs the other stages, be aware of the role of culture in producing and reproducing gender. Contextualizing and situating actions considering these elements could include involving stakeholders with experience and knowledge of how gender is constructed in that setting. In doing this, and to avoid leveraging or ignoring existing gender roles, norms, and relations to achieve intervention goals, and be more gender responsive, interventions could for example, seek ways to engage one sex in areas that are traditionally associated with the other; and/or implement measures to counter for possible unintended consequences and effects during and after the intervention.

From inception to completion, collect, interpret, and report data by sex and gender. Guidelines for integration of sex and intersectional gender into data collection, analysis, and reporting exist [31,112], as well as studies providing foundational concepts to understand how sex and gender concepts differ and overlap [24,25,28,32].

Along all phases of the intervention, embrace gender responsive communication practices. Tools in different languages exist [113–115] that include guidelines for the use gender neutral language when appropriate [116], and suggestions to avoid using gender-biased language or gender stereotyping in images, narrative, words and quantitative data. Examples of gender responsive wording include: Instead of referring to ‘manpower’ use staffing, workforce or labor; avoid using ‘man’ to refer to human beings or humanity; and instead of using ‘man and wife’ use partners, husband and wife, or wife and husband.

### Strengths and limitations of this study

Generalizing findings from a sample of 20 social marketing interventions should be made with caution. Including other types of interventions would provide a larger sample and broader understanding of the extent to which gender is incorporated into interventions addressing NTDs. Nonetheless, the data provide valuable insights about the gender responsiveness of social marketing interventions.

Using the GAT posed limitations related to its design, some of which were addressed by modifying the tool, using the intersectional sex and gender search, and by doing a qualitative assessment of the results. The GAT was not designed to determine the specific gender approach applied. Consequently, this study did not classify the interventions according to these five approaches. The original tool includes yes/no responses and ends with classifying an intervention as responsive or not. In this study, we added the not available response option to report the absence of data, and documented texts supporting the response chosen.

Furthermore, the tool does not capture the use of concepts, non-binary sex, and gender, nor the intersectional characteristic of gendered experiences. For this reason, additional data were coded. Another limitation was the lack of guidelines about how to assess cultural aspects that shape context-specific gender dimensions. This study tried to acknowledge this by reporting the countries where the interventions were implemented and the particularities found. Future studies using the GAT would benefit from further modifications to the tool or from redesigning it, for example, by adding questions to help identify the specific gender approach used by each intervention. It would enable identifying more specific characteristics that make interventions gender responsive or not.

Finally, findings should be viewed understanding that they are influenced by the own historical and cultural positionalities of the researchers involved, who understand gender based on western and Latin American conceptions of the interplay of intersectional gender and sex.

## Conclusion

In 1995 governments joined efforts to address gender inequality when signing the Beijing Declaration and Platform for Action [2]. This commitment was also visible in global policies such as the Millennium Declaration [117], and more recently with the 2030 Agenda for Sustainable Development [1]. Although the importance of gender in NTDs has been raised [29,48,50,118], the current NTDs global guiding documents [119,120] lack sex and gender considerations. Conversations to develop a new NTD Roadmap promise to open a window of opportunity to integrate gender prominently into the NTD agenda [29]. This is important given the findings of this study that show there is much to be improved to achieve gender responsive health interventions. Our findings highlight that most social marketing interventions addressing NTDs are not gender responsive, in part due to the lack of reporting. This lack of evidence is one of the main obstacles to inform gender responsive policies for NTDs, and hinders ‘moving from theory and research to policy and action’ [121]. Interventions developers should commit to gender equality; not ignore the gender order of the setting in which they are intervening; collect, interpret and report data by sex and gender, embrace gender responsive communication; and be aware of the cultural aspects of gender. In doing so, interventions will have much greater potential to lessen the negative effects of gender inequities and inequalities that restrict reaching health outcomes.

## Paper context

Little is known about how social marketing interventions focusing on neglected tropical diseases have included gender in their design or implementation. To our knowledge, this is the first time that the gender responsiveness of social marketing interventions addressing NTDs is assessed. The WHO Gender Assessment Tool was used, together with qualitative data, to provide an insight into the state of gender integration in social marketing interventions on NTDs.
